# Veterinary Medical Association of New York County

**Published:** 1895-06

**Authors:** J. E. Ryder

**Affiliations:** Secretary


Secretary.

### VETERINARY MEDICAL ASSOCIATION OF NEW YORK COUNTY.

THE regular meeting was held at the New York College of Veterinary Surgeons, on Tuesday, May 7, 1895, at 8.30 o'clock, the President, Dr. Huidekoper, in the chair.

On roll-call the following members responded to their names, viz., Drs. Bretherton, Caulfield, Dickson, Delany, Ferster, S. S. Field, H. S. Field, Giffen, Gill, Glover, Huidekoper, Hanson, Lellman, Machan, Neher, Ryder, Richards, Sherwood, Sielman, Turner, Wolters, and Wellner.

The minutes of last meeting were read and adopted.

The chairman of the Board of Censors, reported favorably the name of Dr. M. O. M. Knott, a graduate of the New York College of Veterinary Surgeons, vouched for by Drs. Giffen and Delaney.

The resignation of Dr. A. Liautard, which was laid over at the last meeting, was then taken up, and it was moved by Dr. Field and seconded by Dr. Giffen, that it be accepted. Carried.

Papers being next in order, Dr. Ferster read a very interesting paper on "Horseshoeing," giving many practical points upon the art (*vide* p. 348).

*Reports of Committees.* Judiciary Committee: Dr. Giffen reported as follows: The Jury Bill had passed both houses and had been signed by the Governor; the bill granting an extension of time for non-graduates to register had been killed in the Senate; the Regents' Bill had passed the Assembly and will be up for third reading in the Senate during the present week. Moved and seconded, that the report be accepted; carried. Moved and seconded, that the expenses incurred by Drs. O'Shea and Huidekoper during their trips to Albany be paid; carried. Moved and seconded, that a vote of thanks be extended to Drs. O'Shea and Huidekoper for their good work at Albany; carried.

Committee on Certificates reported progress.

Committee on Revision of Constitution and By-laws: Dr. Hanson, acting chairman, made the report for the committee. It was then moved and seconded, that each article be taken up separately and acted upon; carried.

ART. IV. Constitution. The annual election shall take place on the evening of the first Tuesday of December; carried.

ART. VI. By-laws. A board of Censors shall be elected by the members at each annual meeting; not adopted.

ART. VII. 1. Charter Members: All those practitioners of veterinary medicine who were present at the first meeting to assist in the organization of the above Association, etc.; adopted.

2. Future Members: Applications for membership shall submit their names upon one of the Association's application blanks, duly vouched for by two members of the Association. The initiation fee shall accompany such application, which is returnable should he not be elected to membership; adopted.

3. A two-thirds vote will be necessary to constitute an election to membership; adopted.

ART. VIII. A member elect shall pay to the Treasurer his annual dues, which shall be five dollars; adopted.

ART. XIII. The Association shall meet upon the first Tuesday of each month, except during July, August, and September; election falling on a Tuesday the meeting will be held the following Thursday; adopted.

ART. XIV. The Judiciary Committee shall consist of five members, elected at the annual meeting; not adopted.

Code of Ethics: No member shall endeavor to build up a practice by undercharging his brother-member; adopted.

Committee on Charges reported progress.

Election of Members: Moved and seconded, that the by-laws be suspended; carried. Moved and seconded, that the Secretary cast a ballot for Dr. Knott; carried. Dr. Knott was then declared a member of the Association.

New Business: Moved and seconded, that the Constitution and By-laws be printed as adopted; carried. Moved and seconded, that a committee of five be appointed as a Reception Committee for the State Meeting to be held in this city in September next, the said committee to report at the next meeting; carried. Meeting adjourned. J. E. RYDER, D.V.S.,

